# Duodenal diverticula perforation caused by an impacted bezoar successfully treated by endoscopic drainage and lithotripsy: A case report and literature review

**DOI:** 10.1002/ccr3.6619

**Published:** 2022-11-20

**Authors:** Takeshi Tadokoro, Koichi Oishi, Yosuke Namba, Tomoaki Bekki, Sho Okimoto, Shoichiro Mukai, Yasufumi Saito, Seiji Fujisaki, Mamoru Takahashi, Teruo Mouri, Toshihiro Nishida, Toshikatsu Fukuda

**Affiliations:** ^1^ Department of Surgery Chugoku Rosai Hospital Kure, Hiroshima Japan; ^2^ Department of Gastroenterology Chugoku Rosai Hospital Hiroshima Japan; ^3^ Department of Pathology Chugoku Rosai Hospital Hiroshima Japan

**Keywords:** duodenal diverticula perforation, endoscopic drainage, endoscopic lithotripsy, phytobezoar

## Abstract

Duodenal diverticula perforation due to an impacted bezoar is a rare disease. Surgical treatment is associated with high rates of complications and mortality; therefore, treatment strategies must be carefully decided. Endoscopic treatment offers significant benefits to patients over surgery.

## Abstract 2

Duodenal diverticula perforation due to an impacted bezoar was performed endoscopic treatment. Endoscopic treatment offers significant benefits to patients over surgery.

## 1 BACKGROUND

The diverticulum of the duodenum is the second most common location after the colon.^1^ Most patients are asymptomatic. However, among complications, duodenal diverticula perforation (DDP) rarely occurs, but when it does, it often perforates the in retroperitoneum. Furthermore, bezoar‐induced DDP in duodenal diverticula is substantially rare.^2^ Duodenal diverticula perforation is the most threatening complication, and appropriate treatments for perforated duodenal diverticula remain unclear, ranging from conservative therapy to surgery, including pancreatoduodenectomy. Surgical intervention may increase morbidity and mortality rates. Therefore, non‐operative management can be considered in some patients with perforated diverticulum who have stable vital signs without generalized peritonitis or in elderly patients with comorbidities.^3^ Recently, several cases of conservative treatment with endoscopy have been reported. We are describing the successful endoscopic drainage and lithotripsy of a DDP associated with an impacted bezoar.

## 2 CASE PRESENTATION

**FIGURE 1:**
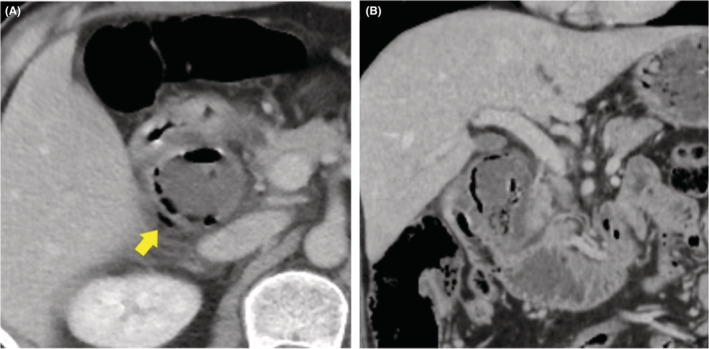
Abdominal computed tomography (CT) (A) axial image, (B) coronal image. CT revealed an enlarged duodenal diverticulum within the second portion of the duodenum and free air in the retroperitoneum (yellow closed arrow)

**FIGURE 2:**
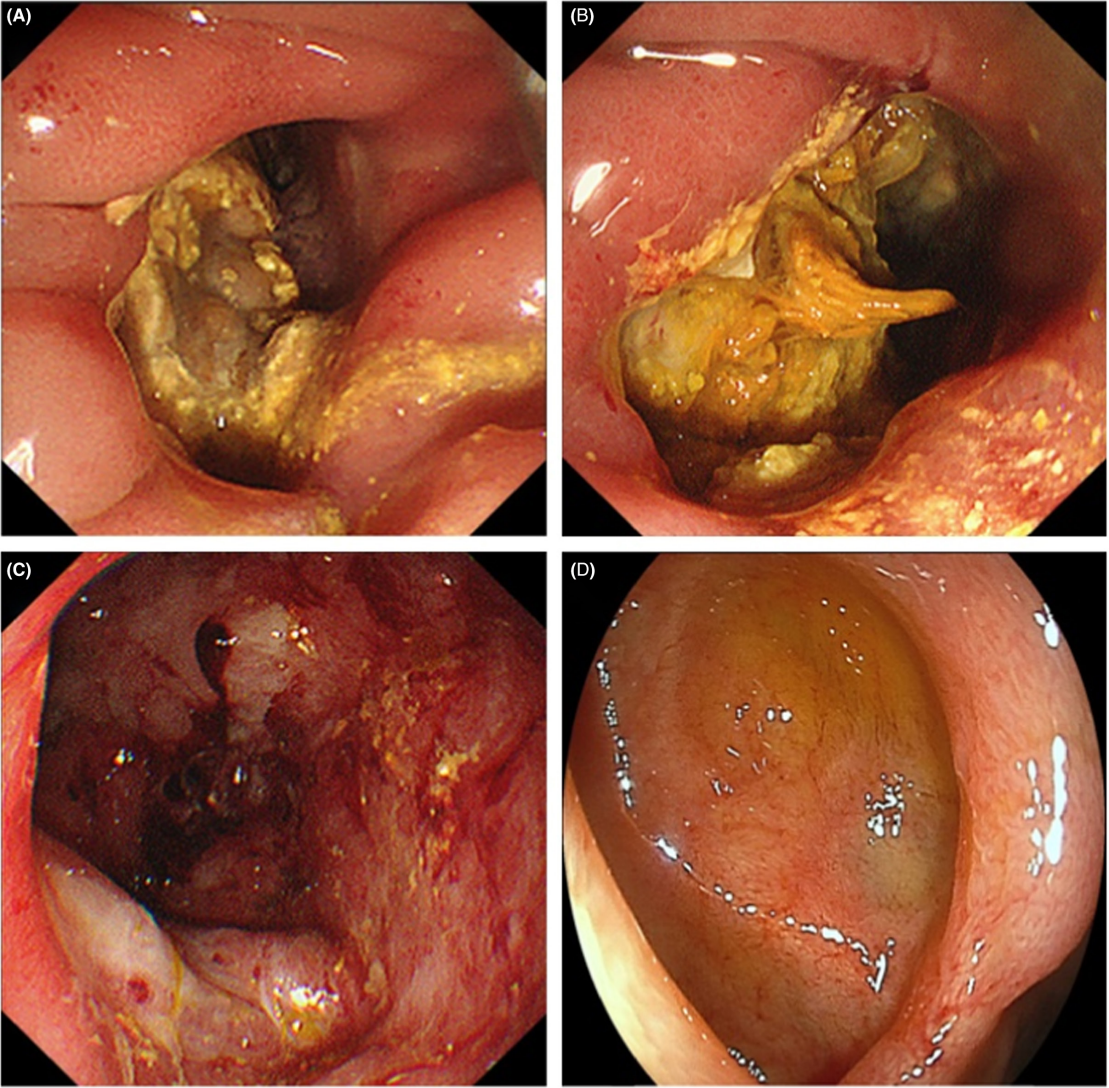
Endoscopic findings. (A) First endoscopy. A duodenal diverticulum on oral side of ampulla of Vater with an impacted large phytobezoar and pus was noted. (B) Second endoscopy. The phytobezoar was slightly softer than that at the previous endoscopy and was incompletely eliminated by crushing with forceps. (C) Third endoscopy. The phytobezoar was removed. (D) Subsequent upper gastrointestinal endoscopy performed after 3 months. The mucosa of the duodenal diverticulum was regenerated

**FIGURE 3:**
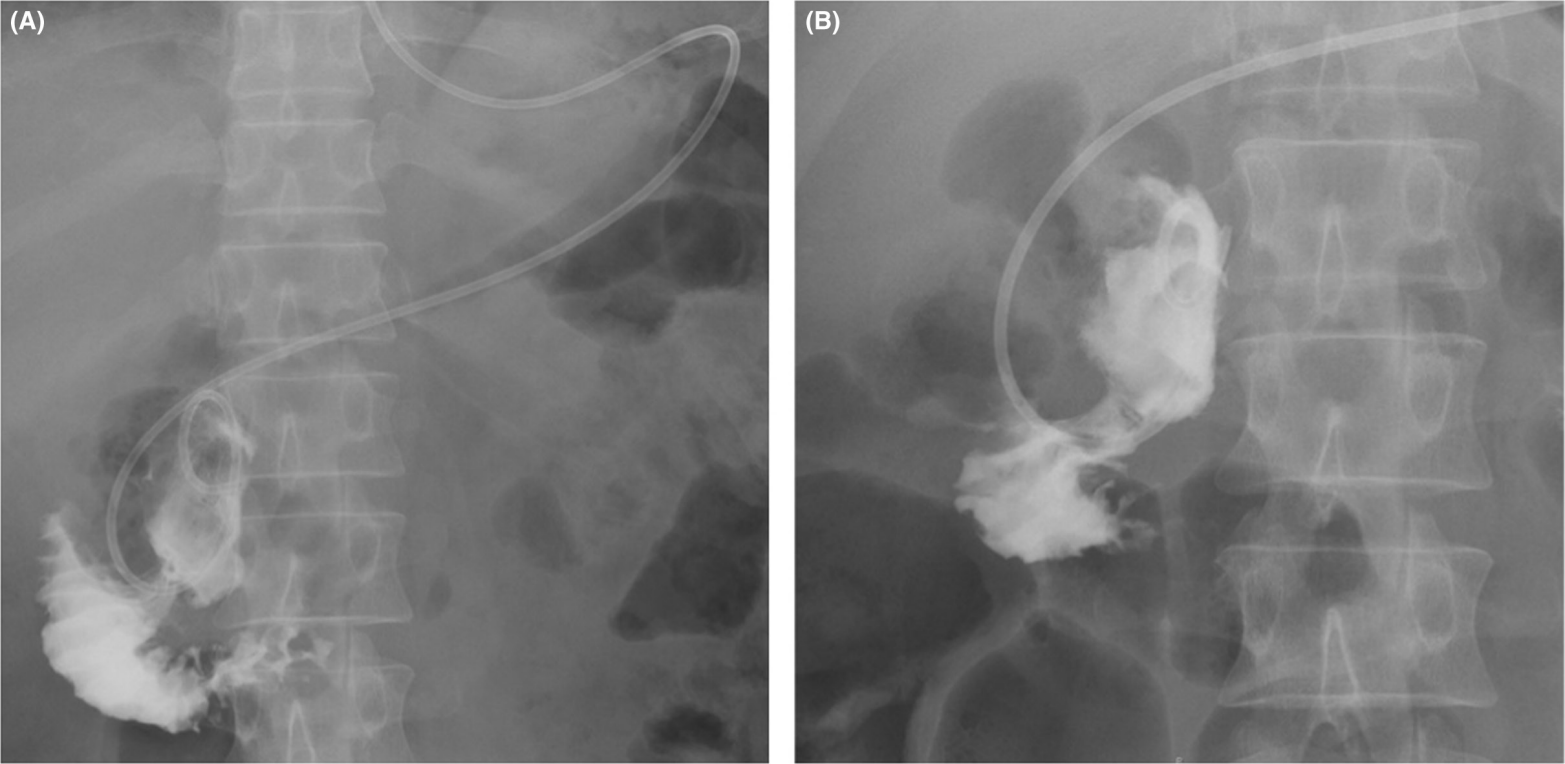
Diverticulogram findings. (A) First diverticulogram. Abscess drainage was performed using a 7.5 Fr endoscopic nasobiliary drainage catheter. (B) Second diverticulogram. A 6Fr ENBD catheter was placed in the cavity again

The 52‐year‐old Japanese man who had undergone laparoscopic surgery for a duodenal ulcer 12 years ago presented with upper abdominal pain. His symptoms persisted from the morning of that day. On arrival at the emergency room, his blood pressure was 166/92 mmHg, pulse was 65 beats per minute, and oxygen saturation was 98% on room air. He experienced mild discomfort, with a body temperature of 39.0°C. No signs of peritoneal irritation were noted. The laboratory data showed a high white blood cell count of 21,200/μl but c‐reactive protein level of 0.3 mg/dl was at normal level (<1.0 mg/dl). Abdominal computed tomography (CT) revealed an expanded duodenal diverticulum inside the second portion of the duodenum and free air in the retroperitoneum outside the diverticulum (Figure 1). Duodenal ulcer perforation was suspected based on the patient's history. However, CT imaging was most likely caused by perforation of a duodenal diverticulitis because past CT showed an existing duodenal diverticulum. Because there were no symptoms of peritoneal irritation, broad‐spectrum antibiotics, placement of a nasogastric tube, and use of proton pump inhibitors (PPIs) were initiated. However, the inflammatory findings did not improve; therefore, endoscopic drainage was performed, on the fourth day of admission. On endoscopy, a duodenal diverticulum on oral side of ampulla of Vater with a fixed large phytobezoar and a large amount of pus from duodenal diverticular, opening size approximately 10 mm, was observed (Figure 2A). Because the bezoar blocked the orifice of the duodenal diverticulum, it perforated the retroperitoneum and became an abscess cavity. The phytobezoar was so large and of hard consistency that endoscopic removal during a single session was difficult. However, we were able to destruct part of the bezoar with forceps, so we thought we could remove it with multiple lithotripsies. First, it needed to flush the abscess, so A 7.5 Fr endoscopic nasobiliary drainage (ENBD) catheter was placed in the duodenal diverticulum. Contrast injection showed a large translucent image of the diverticulum (Figure 3A). The duodenal diverticulum was washed daily with 20 ml of saline from the ENBD catheter. As a result, the fever resolved and inflammatory findings improved. On the eighth day of admission, the second endoscopy was performed. On the second endoscopy, since the phytobezoar was slightly softer than that on the previous endoscopy, it was gradually eliminated by crushing with forceps (Figure 2B). However, part of the phytobezoar remaining deep in the cavity could not be completely removed, so a 6 Fr ENBD catheter was placed in the cavity again (Figure 3B). The following day, saline was injected through the tube to clean the abscess cavity, and the phytobezoar was completely removed by a third endoscopic treatment, on the tenth day of admission (Figure 2C). The day after the third endoscopic treatment, the patient started oral intake, and the forth endoscopy showed that the duodenal diverticular mucosa was tending to heal, on the thirteenth day of admission. On the sixteenth day of admission, the patient was discharged from the hospital without any complications. After 3 months of hospital stay, subsequent upper gastrointestinal endoscopy showed that the cavity of the duodenal diverticulum had shrunk and the mucosa of the diverticulum had regenerated (Figure 2D).

## 3 DISCUSSION

Duodenal diverticula are found in 5–10% of patients undergoing radiological or endoscopic procedures and in 15–23% of patients at autopsy.^1^ The duodenum is the most common site for gastrointestinal diverticula after the colon, especially in the parapapillary region of Vater and the horizontal and ascending portions of the duodenum.^4^, ^5^ Unlike colon diverticulum, duodenal diverticulum is relatively asymptomatic. However, the risk of perforation should be kept in mind.^6^ Most of these perforations were seen within the second portion of the duodenum, mainly along the medial wall, within 2 cm of the ampulla of Vater. Duodenal diverticulitis was the most common cause of DDP, representing 69% of total associated cases.^7^ As perforation often occurs in the retroperitoneum, typical signs of peritonitis are often absent. Due to the lack of pathognomonic signs or symptoms, DDP is often clinically mistaken for acute cholecystitis, appendicitis, and perforated duodenal or gastric ulcers. Shimada et al. evaluated all 202 cases of DDP reported worldwide between 1907 and 2020. A total of 83% of all reported cases underwent surgical treatment.^8^ Simple closure of the perforated site is anatomically difficult when a duodenal diverticulum perforates the retroperitoneum. A pancreaticoduodenectomy must be performed to resect the duodenum that has the perforated site. However, this treatment appears to be highly invasive. Indeed, several cases in which surgical treatment was chosen reportedly involved only drainage for retroperitoneal perforation.^9^, ^10^ In addition, these patients required a longer time to achieve postoperative cure. The morbidity and mortality rate for surgical options are reaching as high as 30%,^11^ including duodenal leak and fistulization; the option of conservative treatment has become more common. The success rate of conservative treatment, initially with broad‐spectrum antibiotics, is very low. Given these facts, nonsurgical drainage from a retroperitoneal abscess is an option for treating perforated diverticula. However, percutaneous drainage can be technically challenging. Therefore, if the symptoms improve with endoscopic drainage, it is a valuable option.

Bezoars are composed of vegetable matter (phytobezoar), hair (trichobezoar), or other unusual materials. Previous gastric surgery (disturbance of pyloric function, gastric emptying, and hypoacidity), poor mastication, or overindulgence with foods with high fiber content are common predisposing factors for bezoar formation. There is only one report of laparoscopic resection of a bezoar in a duodenal diverticulum,^12^ but duodenal diverticular perforation due to phytobezoar is rare.

We used the keywords “DDP” and “Endoscopic treatment” to conduct a PubMed search, there were several reports of endoscopic treatment of DDP. The information regarding the reported 7 patients and our case is summarized in Table 1.^10^, ^13^, ^14^, ^15^, ^16^, ^17^ Endoscopic treatment of DDP has increased since 2015. An ENBD catheter,^17^ stent,^15^ and endoscopic negative pressure^16^ were used for treatment. In all the cases, antibiotics were administered on admission, and endoscopic treatment was performed concurrently or secondarily. These reports demonstrated food debris and enteroliths in the duodenal diverticulum, which were removed using lithotomy^10^ or a combination of a balloon catheter, Dormia basket, and an endoscopic retrograde cholangiopancreatography (ERCP) injection catheter.^13^ For lithotripsy, tools were selected for each case. Complete removal was achieved in all reported cases. The abscess was treated with a stent, ENBD catheter, and ENPT. The stent was not suitable for this case because it could not be cleaned and could only be removed endoscopically. The symptoms improved immediately after endoscopic treatment. In this case, the retroperitoneal abscess was cleaned with the placement of an ENBD catheter in the diverticulum and endoscopic lithotripsy and was significantly effective. However, the bezoar was so hard and sticky that three endoscopic treatments were required to remove it. Flushing the ENBD catheter with saline was particularly useful because now the bezoar was smaller and softer. It has been reported that DDP can sometimes be relieved with fasting and antibiotics. However, in this case, there was no improvement in inflammatory findings or abdominal symptoms after starting antibiotics; therefore, endoscopy was performed. Endoscopic treatment was effective because the patient's condition did not improve until the phytobezoar of the duodenal diverticulum was removed. Endoscopy can provide a more appropriate diagnosis, drainage, tube washing, and even stone removal. Endoscopic therapy, due to its therapeutic diversity, is valuable for the treatment of DDP.

**TABLE 1:** Review of endoscopic treatment for duodenal diverticula perforation

Case	Author		Year	Age	Sex	Symptoms	Contents other than pus in duodenal diverticula	Endoscopic treatment	Hospital stay (days)
1	Tsukamoto T	[10]	1999	66	F	fever and tenderness with rebound in the right upper quadrant	An enterolith	endoscopic lithotomy	NA
2	Eeckout G	[13]	2000	49	F	epigastric pain, nausea, and vomiting	Impacted with food debris	Balloon catheter, dormia basket, and ERCP injection catheter	NA
3	Sasaki F	[14]	2015	58	M	abdominal and back pain	None	endoscopic tissue shielding using polyglycolic acid sheets and fibrin glue	NA
4	Shirobe T	[15]	2017	52	F	pain at right hypochondriac region	A bilirubin calculus	Placed stents in the bile duct and retroperitoneal abscess cavity	22
5	Wichmann D	[16]	2021	82	F	NA	None	ENPT	20
6	Wichmann D	[16]	2021	69	F	NA	None	ENPT	20
7	Kawano S	[17]	2022	70	M	abdominal pain	An incarcerated enterolith	ENBD	35
8	Our case			52	M	upper abdominal pain and fever	An impacted phytobezoar	ENBD and endoscopic lithotripsy	15

Abbreviations: ENBD, endoscopic nasobiliary drainage; ENPT, endoscopic negative pressure therapy; ERCP, endoscopic retrograde cholangiopancreatography; F, female; M, male; NA, not available.

In the current case, a previous CT scan revealed a duodenal diverticulum, which helped in the diagnosis of DDP. It remains difficult to distinguish DDP from duodenal perforation based on image studies and clinical findings. However, even if the presence of a duodenal diverticulum was not evident and CT showed a localized fluid collection around the duodenum and no peritoneal irritation symptoms, DDP should be suspected and endoscopic treatment considered.

## 4 CONCLUSION

This is a rare case of retroperitoneal perforation of a duodenal diverticulum due to a bezoar impaction. Endoscopic treatment was successful, and surgery was avoided. The endoscopic approach is minimally invasive and offers significant advantages when perforation is limited to a retroperitoneal abscess. Endoscopic drainage and lithotripsy should be considered for DDP caused by bezoars.

## AUTHOR CONTRIBUTIONS

TT and KO analyzed interpreted data and were major contributors in writing the manuscript. All authors read and approved the final manuscript.

## CONFLICT OF INTEREST

The authors have no conflicts of interest to declare.

## ETHICAL APPROVAL

This manuscript conforms to the provisions of the Declaration of Helsinki of 1995 (as revised in Brazil in 2013).

## CONSENT

Written informed consent was obtained from the patient to publish this report in accordance with the journal's patient consent policy.

## Data Availability

The datasets supporting the conclusions of this article are included within the article.

## References

[1] Degheili JA , Abdallah MH , Haydar AA , Moukalled A , Hallal AH . Perforated duodenal diverticulum treated conservatively: another two successful cases. Case Rep Surg. 2017;2017:4045970. 28555171 10.1155/2017/4045970 PMC5438833

[2] Kim JH , Chang JH , Nam SM , et al. Duodenal obstruction following acute pancreatitis caused by a large duodenal diverticular bezoar. World J Gastroenterol. 2012;18:5485‐5488. 23082068 10.3748/wjg.v18.i38.5485 PMC3471120

[3] Oukachbi N , Brouzes S . Management of complicated duodenal diverticula. J Visc Surg. 2013;150:173‐179. 23810155 10.1016/j.jviscsurg.2013.04.006

[4] Duarte B , Nagy KK , Cintron J . Perforated duodenal diverticulum. Br J Surg. 1992;79:877‐881. 1422745 10.1002/bjs.1800790907

[5] Volchok J , Massimi T , Wilkins S , Curletti E . Duodenal diverticulum: case report of a perforated extraluminal diverticulum containing ectopic pancreatic tissue. Arch Surg. 2009;144:188‐190. 19221332 10.1001/archsurg.2008.532

[6] Song S . Management of perforated duodenal diverticulum: report of two cases. Korean J Gastroenterol. 2015;66:159‐163. 26387699 10.4166/kjg.2015.66.3.159

[7] Thorson CM , Paz Ruiz PS , Roeder RA , Sleeman D , Casillas VJ . The perforated duodenal diverticulum. Arch Surg. 2012;147:81‐88. 22250120 10.1001/archsurg.2011.821

[8] Shimada A , Fujita K , Kitago M , et al. Perforated duodenal diverticulum successfully treated with a combination of surgical drainage and endoscopic nasobiliary and nasopancreatic drainage: a case report. Surg Case Rep. 2020;6:129. 32514821 10.1186/s40792-020-00891-0 PMC7280391

[9] Tsukamoto T , Hasegawa I , Ohta Y , et al. Perforated duodenal diverticulum caused by enterolith. Nihon Shokakibyo Gakkai Zasshi. 1998;95:895‐899. 9752700

[10] Tsukamoto T , Ohta Y , Hamba H , et al. Perforated duodenal diverticulum: report of two cases. Hepatogastroenterology. 1999;46:1755‐1758. 10430338

[11] Teven CM , Grossman E , Roggin KK , Matthews JB . Surgical management of pancreaticobiliary disease associated with juxtapapillary duodenal diverticula: case series and review of the literature. J Gastrointest Surg. 2012;16:1436‐1441. 22392090 10.1007/s11605-012-1856-z

[12] Pergel A , Yucel AF , Aydin I , Sahin DA . Laparoscopic treatment of a phytobezoar in the duodenal diverticulum ‐ report of a case. Int J Surg Case Rep. 2012;3:392‐394. 22659120 10.1016/j.ijscr.2012.03.020 PMC3376636

[13] Eeckhout G , Vanstiphout J , Van Pottelbergh I , et al. Endoscopic treatment of a perforated duodenal diverticulum. Endoscopy. 2000;32:991‐993. 11147951 10.1055/s-2000-9615

[14] Sasaki F , Kanmura S , Nasu Y , et al. Double‐balloon enteroscopy‐assisted closure of perforated duodenal diverticulum using polyglycolic acid sheets. Endoscopy. 2015;47 Suppl 1 UCTN:E204‐E205. 26062149 10.1055/s-0034-1391651

[15] Shirobe T , Kawakami H , Abe S , Yokochi T . Retroperitoneal perforation arising from duodenal diverticulum treated by endoscopic drainage: a case report. Clin Case Rep. 2017;5:654‐657. 28469870 10.1002/ccr3.921 PMC5412815

[16] Wichmann D , Jansen KT , Onken F , et al. Endoscopic negative pressure therapy as stand‐alone treatment for perforated duodenal diverticulum: presentation of two cases. BMC Gastroenterol. 2021;21:436. 34802417 10.1186/s12876-021-02018-7 PMC8607673

[17] Kawano S , Tsuchiya Y , Motegi S , et al. Management of a perforated duodenal diverticulum using an endoscopic nasobiliary drainage tube: a case report. Nihon Shokakibyo Gakkai Zasshi. 2022;119:47‐52. 35022370 10.11405/nisshoshi.119.47

